# A case of intradural lumbar disc herniation

**DOI:** 10.1002/ccr3.7514

**Published:** 2023-06-13

**Authors:** Utsav Bhattarai, Pritam Gurung, Janam Shrestha, Sudan Dhakal, Samir Acharya, Basant Pant

**Affiliations:** ^1^ Department of Neurosurgery Annapurna Neurological Institute and Allied Sciences Kathmandu Nepal

**Keywords:** disc, intradural, lumbar, spine

## Abstract

**Key Clinical Message:**

MRI remains the best tool in the diagnosis of this disease entity however preoperative diagnosis remains a difficult task. A high degree of suspicion is raised when intraoperative findings and preoperative image description become incompatible.

**Abstract:**

Lumbar disc herniation into the dural space is a rare phenomenon of lumbar disc degeneration with an unclear remaining pathogenesis. Intraoperative ultrasonography and histopathological examination of resected specimen help in the diagnosis of intradural disc herniation. Prompt surgery is recommended due to the high incidence of cauda equina syndrome.

## Graphical abstract

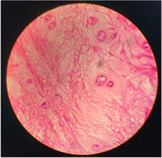

## 1 INTRODUCTION

Lumbar disc herniation into the dural space is a very rare phenomenon of degenerative lumbar disc disease concerning its uncertain pathology with L4‐L5 and L5‐S1 levels being the most common region of involvement.^1^ Magnetic resonance imaging (MRI) remains the best tool in the diagnosis of this disease's entity. However, preoperative diagnosis remains a difficult task. In this report, we describe a patient who underwent the surgical resection of an intradural disc herniation.

## 2 CASE PRESENTATION

**FIGURE 1:**
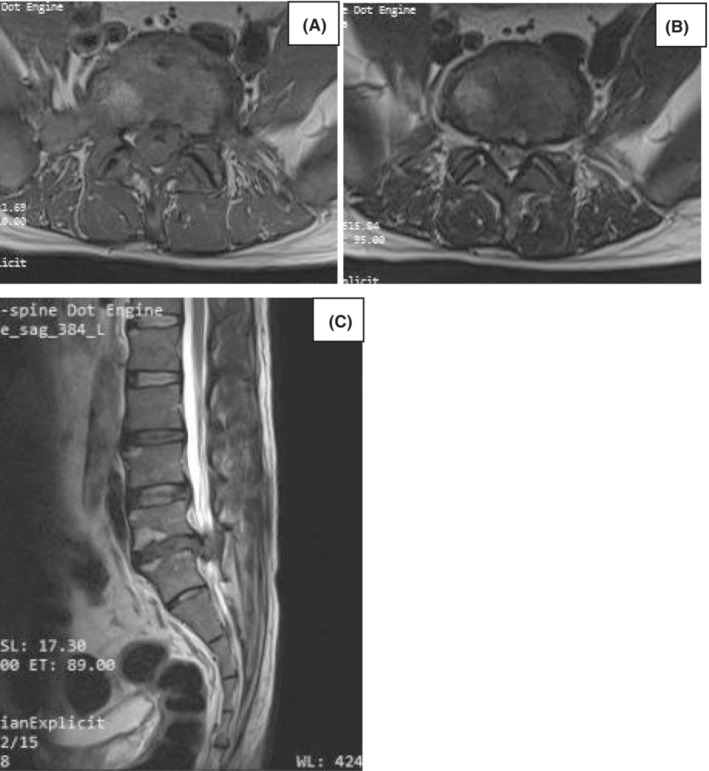
MRI lumbar T1 & T2 axial and T2 sagittal revealed focal posterocentral disc bulge with central and B/l paracentral extrusion of the disc elements in the superior and inferior spinal canal at L5‐S1 level causing severe narrowing of the central spinal canal and compression of the descending nerve roots as well as traversing S1 left nerve root.

A 43‐year‐old man presented to our emergency room (ER) with the complaint of left‐sided lower back pain radiating to the left lower limb for the past 2 years. Increased severity was described for the last 10 days with weakness in his left lower limb, and history of urinary incontinence (overflow) since the last 2 days. The personal history showed no diagnosis of hypertension or diabetes mellitus. No relevant familial or psychosocial history was reported. Performing the straight leg raising test, both legs were found to be at around 60 degrees of possible elevation. The extensor hallucis longus (EHL) and flexor hallucis longus (FHL) force test showed a strength of 3/5 on both sides. Sensory examination showed perineal and anterolateral right foot hypoesthesia. Performed MRI of the lumbo sacral spine revealed diffuse bulging of the disc with a left postero central and sub articular extrusion at L5‐S1 level, as well as thecal compression, and impingement of the left traversing S1 nerve root (Figure 1A–C). A presumptive diagnosis of a herniated lumbar disc with radiculopathy and cauda equina syndrome was made based on the clinical evaluation of the patient.

On examination, the Oswestry Disability Index (ODI) was found to be 92%.^2^

**FIGURE 2:**
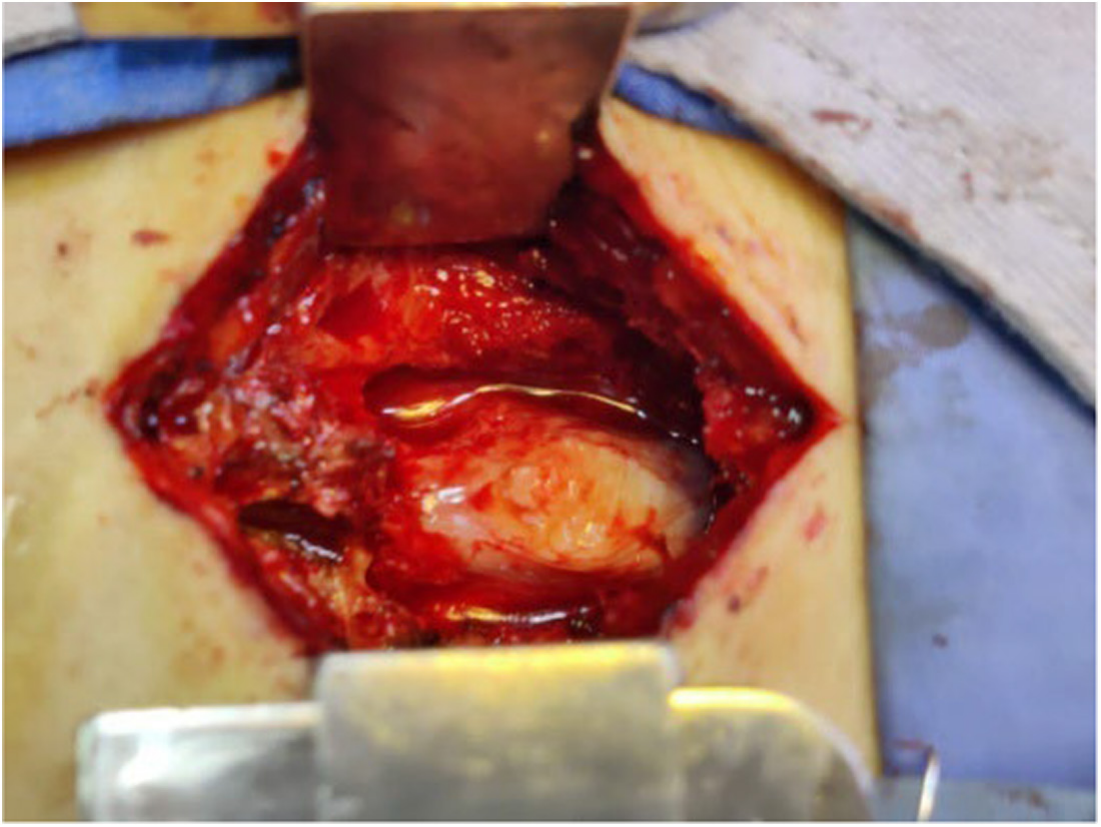
Intraoperative image showing tense and bulging mass inside the thecal sac at L5‐S1 level.

**FIGURE 3:**
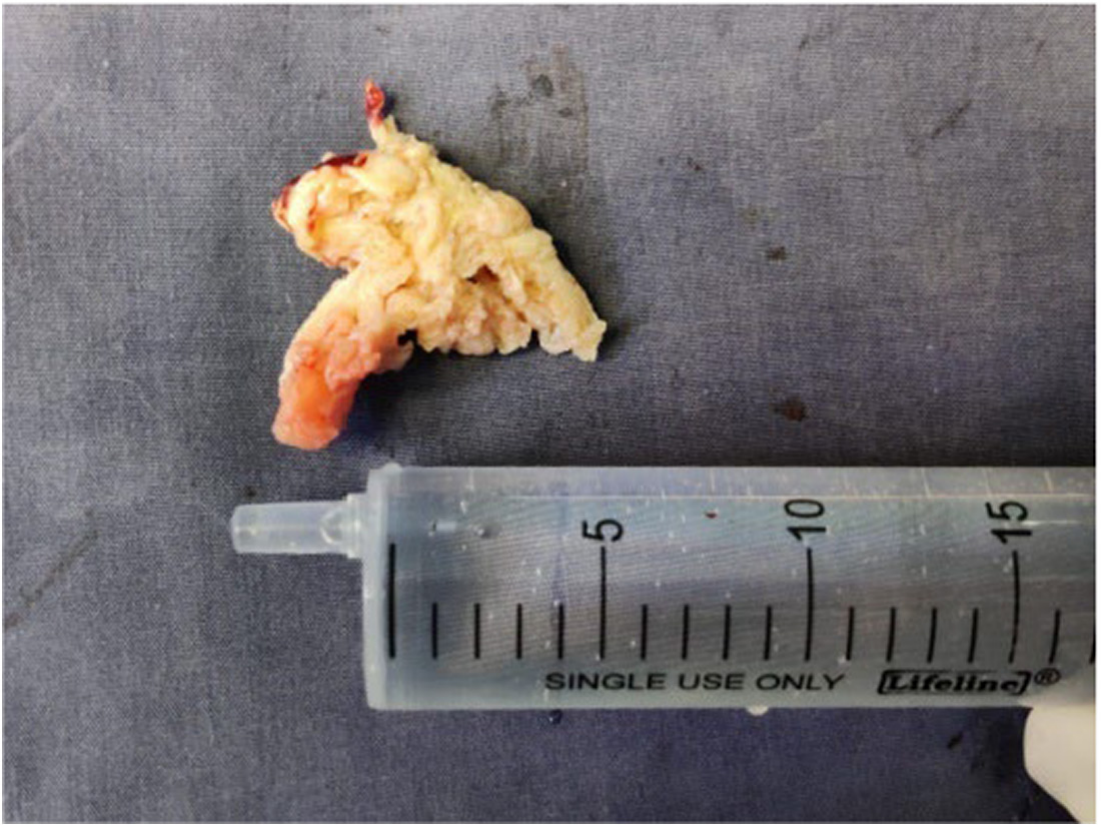
After removal a hard fungating mass similar to tissue texture of nucleus pulposus was revealed.

**FIGURE 4:**
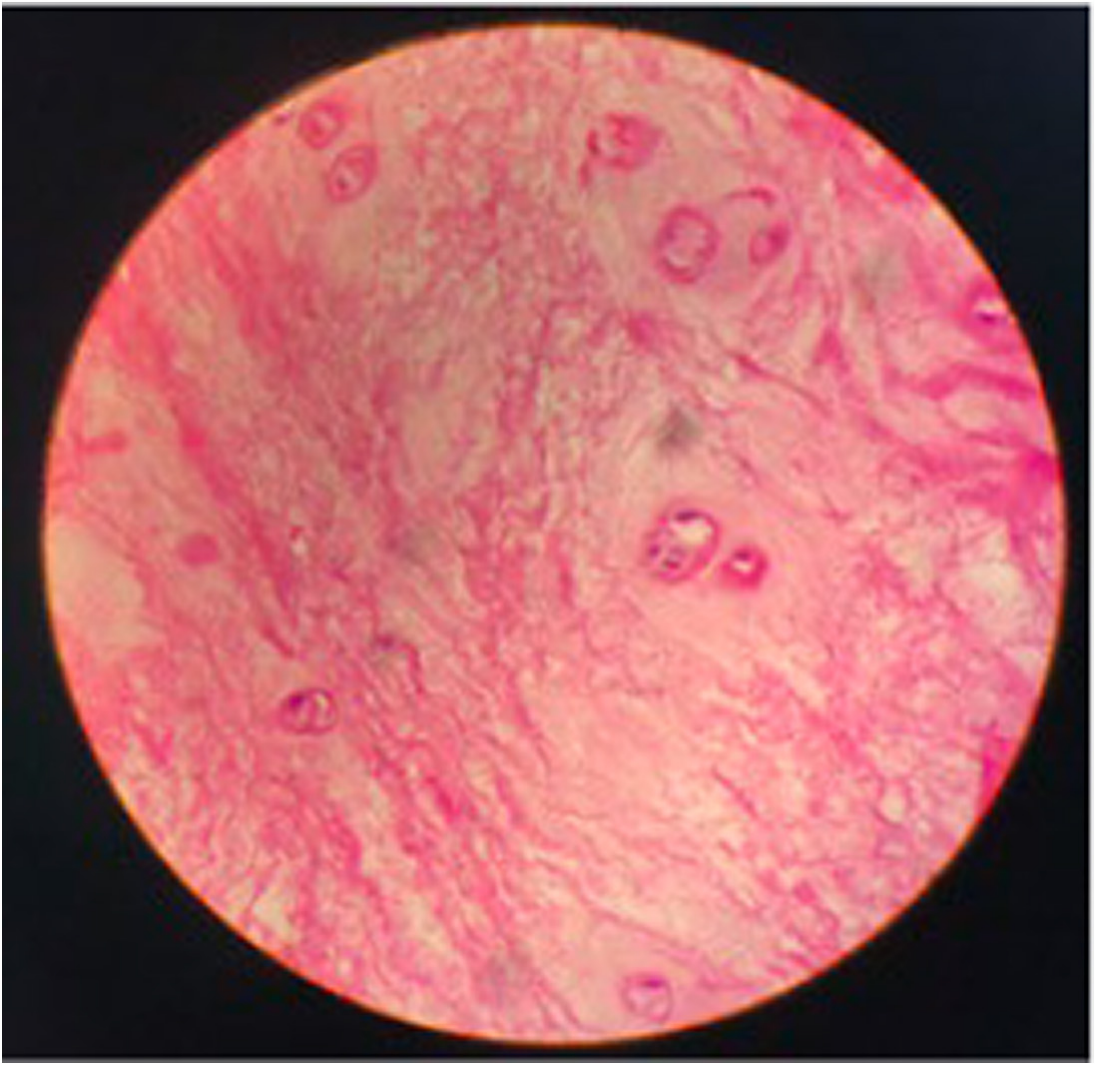
Histopathological examination showing disc tissue.

The patient underwent total laminectomy at the level of L5‐S1. The disc was successfully removed from both sides of the shoulder end of the nerve root. Additionally, foraminotomy was bilaterally performed. Intraoperatively only minimal disc fragments were found over the epidural space. The dorsal dura mater showed tension and bulging at L5‐S1 level (Figure 2). Subsequently, dorsal durotomy was performed revealing a hard fungating mass over the arachnoid membrane with a tissue similar to the texture of nucleus pulposus (Figure 3). The mass was dissociated and revelaed itself during durotomy. Followed histology report confirmed it to be disc tissue (Figure 4).

Post operatively, the patient's left lower back pain and left leg radiculopathy along with the left lower limb weakness and numbness improved significantly. In addition, the patient also showed immediate cessation of urinary incontinence. The follow up at 1 and 3 months of surgery showed, significant improvement in the patient's symptoms with an ODI score of 10 and 6, respectively. The improvement is mirrored in the patient's abiltity to cope with most daily activities after the procedure. No further treatment is indicated apart from advice on lifting, sitting and exercise.

## 3 DISCUSSION

Intradural disc herniation (IDH) is a rare phenomenon, which accounts for only 0.25%–0.35%^3^ of all disc herniations with the lumbar region being the most common site of occurrence.^4^ The most frequently affected level is L4‐L5.^5^ Since IDH is an uncommon condition, other spinal pathologies such as neurofibroma, lipoma, meningioma, epidermoid tumor, arachnoid cyst, archnoiditis and metastasis should be considered as differential diagnosis.^5^ Given that IDH can mimic an intradural tumor^6^; MRI will help with clarification. On MRI, there is poor visualization on T1‐weighted imaging and whorl‐like mixed intensity sequence on T2‐weighted image, as well as marked ring enhancement following the administration of gadolinium.^7^

Males are more often prone to the entity of IDH than females. Average age of onset is between 50 and 60 years. Symptoms of IDH may vary depending on the location of the herniated disc, with cervical IDH patients presenting clinical findings of the Brown Sequard Syndrome.^8^, ^9^ Patients with involvement of the lumbar region may show symptoms of incomplete and transient quadriparesis presenting with long history of radiculopathic pain with acute presentation matching symptoms of cauda equina syndrome.^5^ In this case, both radiculopathy and cauda equina symptoms gradually improved post operatively. As mentioned earlier there was cessation of incontinence postoperatively; however, he had to strain while initiation of micturition for which physiotherapy and acupuncture therapy were provided to the patient.

The pathogenesis of IDH remains unclear. Few theories have been stated. Several reports suggest that certain factors such as reduced epidural space, resulting from congenital or iatrogenic narrowing of the spinal canal or adhesions between annulus fibrosus, the posterior longitudinal ligament (PLL) and the ventral dura matter may be responsible for IDH.^10^, ^11^ The pathogenetic adhesion between PLL and ventral dura matter was considered to be an important factor to consider.^12^, ^13^ Tateiwa^14^ stated that trauma, previous spine surgery or congenital factors like dural thickness were responsible for adhesions. Floeth and Herdmann^15^ reported that chronic inflammation, as a result of degenerative disc pathologies, favors the development of adhesions and leads to an erosional process following the thinning of the dura. The dura mater and the PLL are in closest proximity at the L4‐L5 level which explains the highest incidence of IDH at the L4‐L5 level.^16^ In this case, dense adhesion between PLL and ventral dura was found bringing up the speculation of a previous history of trauma with disc degeneration resulting in chronic inflammation. Hence, during this process of long term irritation, inflammation together with a sudden force caused the prolapsed disc through the dural erosion into the intradural space.

A preoperative assumption and diagnosis of IDH is difficult. Lesions on imaging might remain unseen or can easily be mistaken for other findings. Definite diagnosis is made only during surgery and not via imaging alone. In this case, the MRI did not indicate the presence of intradural disc fragments, therefore, diagnosis was made intraoperatively.

Intraoperative ultrasonography is helpful in detecting foreign bodies in the dura mater.^17^ If intraoperative ultrasound is used, the lesion can be visualized while the dura is still intact and the dural opening can precisely be tailored to the size and location of the lesion.^18^ Ultrasound provides superior soft tissue imaging of intradural structures compared to MRI. However, in this case we were not able to use ultrasound due to some technical problems that we faced in operation during that time.

The dissection of the dural sheath in the anterolateral portions usually is a difficult step during surgery. Adhesions between the ventral dural sheath and the PLL are extremely resistant, unable to be separated using a sharpless dissection.^19^ In addition, the repair of the anterior dural tear is a difficult procedure. In this case an occlusion with hemostatic material was used. There was no cerebrospinal fluid leak in this case. Prompt surgery has been recommended by most surgeons.^1^, ^20^, ^21^ The outcome of the surgery was found to be closely linked to the preoperative duration of the neurologic symptoms.^21^ Early surgery remains recommended due to the high incidence of cauda equina syndrome.^22^

## 4 CONCLUSIONS

A high degree of suspicion of dural disc herniation is raised when intraoperative findings and preoperative description of image are incompatible. Intraoperative ultrasonography and histopathological examination of resected specimen additionally help in the diagnosis of dural disc herniation. Prompt surgery is recommended because of the high incidence of cauda equina syndrome.

## AUTHOR CONTRIBUTIONS

**Utsav Bhattarai:** Conceptualization; writing – original draft; writing – review and editing. **Pritam Gurung:** Supervision. **Janam Shrestha:** Writing – review and editing. **Sudan Dhakal:** Writing – review and editing. **Samir Acharya:** Writing – review and editing. **Basant Pant:** Supervision.

## FUNDING INFORMATION

None.

## CONFLICT OF INTEREST STATEMENT

None of the authors have potential conflicts of interest to be disclosed.

## ETHICS STATEMENT

Ethical approval of the case report is not needed by the local ethical guideline.

## CONSENT

Written informed consent was obtained from the patient to publish this report in accordance with the journal's patient consent policy.

## Data Availability

Data available on request from the authors.
